# Neutron crystallographic refinement with *REFMAC*5 from the *CCP*4 suite

**DOI:** 10.1107/S2059798323008793

**Published:** 2023-11-03

**Authors:** Lucrezia Catapano, Fei Long, Keitaro Yamashita, Robert A. Nicholls, Roberto A. Steiner, Garib N. Murshudov

**Affiliations:** a Randall Centre for Cell and Molecular Biophysics, Faculty of Life Sciences and Medicine, King’s College London, London SE1 9RT, United Kingdom; b Structural Studies, MRC Laboratory of Molecular Biology, Francis Crick Avenue, Cambridge CB2 0QH, United Kingdom; c Department of Biomedical Sciences, University of Padova, Via Ugo Bassi 58/B, 35131 Padova, Italy

**Keywords:** neutron macromolecular crystallography, crystallographic refinement, H atoms, *REFMAC*5, *CCP*4

## Abstract

Hydrogen (H) atoms are abundant in macromolecules and often play critical roles in enzyme catalysis, ligand-recognition processes and protein–protein interactions. However, their direct visualization by diffraction techniques is challenging. Macromolecular X-ray crystallography affords the localization of only the most ordered H atoms at (sub-)atomic resolution (around 1.2 Å or higher). However, many H atoms of biochemical significance remain undetectable by this method. In contrast, neutron diffraction methods enable the visualization of most H atoms, typically in the form of deuterium (^2^H) atoms, at much more common resolution values (better than 2.5 Å). Thus, neutron crystallography, although technically demanding, is often the method of choice when direct information on protonation states is sought. *REFMAC*5 from the *Collaborative Computational Project No*. 4 (*CCP*4) is a program for the refinement of macromolecular models against X-ray crystallographic and cryo-EM data. This contribution describes its extension to include the refinement of structural models obtained from neutron crystallographic data. Stereochemical restraints with accurate bond distances between H atoms and their parent atom nuclei are now part of the *CCP*4 Monomer Library, the source of prior chemical information used in the refinement. One new feature for neutron data analysis in *REFMAC*5 is refinement of the protium/deuterium (^1^H/^2^H) fraction. This parameter describes the relative ^1^H/^2^H contribution to neutron scattering for hydrogen isotopes. The newly developed *REFMAC*5 algorithms were tested by performing the (re-)refinement of several entries available in the PDB and of one novel structure (FutA) using either (i) neutron data only or (ii) neutron data supplemented by external restraints to a reference X-ray crystallographic structure. Re-refinement with *REFMAC*5 afforded models characterized by *R*-factor values that are consistent with, and in some cases better than, the originally deposited values. The use of external reference structure restraints during refinement has been observed to be a valuable strategy, especially for structures at medium–low resolution.

## 1 Introduction

Knowledge of protonation states and hydrogen (H) atom positions in macromolecules can be critical in helping to formulate functional hypotheses and, generally, in providing a more complete characterization of the biological processes under investigation. H atoms are responsible for the reversible protonation of active site residues involved in enzymatic reactions (Ahmed *et al*., 2007; Fisher *et al*., 2012; Wan *et al*., 2015). They are also necessary for the formation of hydrogen bonds that stabilize macromolecular structures, contributing to the establishment of biological interfaces (Engler *et al*., 2003; Niimura *et al*., 2004; Oksanen *et al*., 2017). Additionally, as H atoms are often involved in determining specificities in protein–ligand recognition processes, their identification and localization may help in the development and design of new therapeutics (Combs *et al*., 2020; Kovalevsky *et al*., 2020; Kneller *et al*., 2022).

The positions of many H atoms in macromolecules can be estimated using the coordinates of their parent atoms (those to which they are covalently bound) and known geometric properties (Sheldrick & Schneider, 1997). This is the case, for example, for amide H atoms in the protein backbone, for those bound to C*^α^* atoms, for those attached to aromatic C atoms *etc*. However, many H atoms of biochemical interest, for example those on the side chains of histidines, protonated aspartates and glutamates, or those associated with multiple favourable positions (the hydroxyl groups of the amino acids serine, threonine and tyrosine), cannot be located on the basis of simple geometric considerations, but need to be determined experimentally (Fisher *et al*., 2009; Gardberg *et al*., 2010).

**Figure 1:**
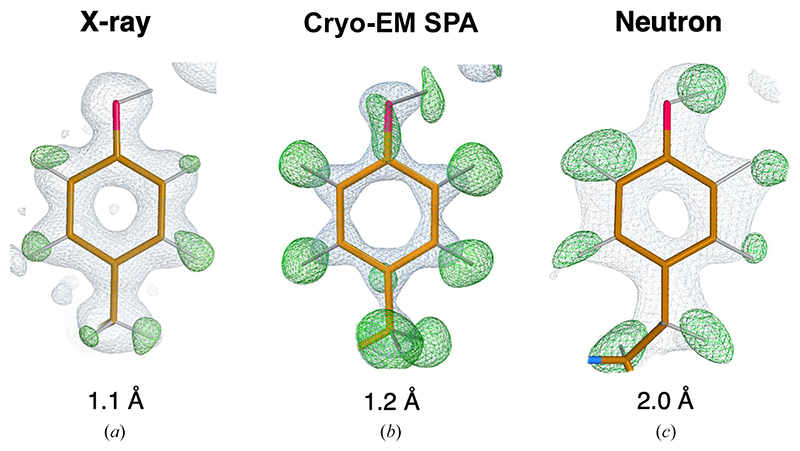
Map examples. (*a*) Electron density maps for Tyr12 (PDB entry 3kyu, 1.1 *Å* resolution) show positive peaks for all aromatic H atoms; however, the H atom on the hydroxyl group is not visible. (*b*) Cryo-EM maps for Tyr32 (PDB entry 7a4m, 1.22 Å resolution) show positive difference peaks for all H atoms. (*c*) Neutron scattering length density maps for Tyr146 (PDB entry 1cq2, 2.0 Å resolution) show positive difference peaks for all H atoms (in the form of ^2^H), including the H atom on the hydroxyl group. Electron and neutron scattering length density maps were calculated using *REFMAC*5 (Murshudov *et al*., 2011), contoured at the +1.0*σ* (2*mF_o_ — DF_c_* in grey) and +3.0*σ (mF_o_ — DF_c_* in green) levels. Cryo-EM weighted and sharpened *F_o_* (grey) and omit (*F*_o_ – *F*_c_, green) maps were calculated using *Servalcat* (Yamashita *et al*., 2021) and contoured at the +1.5*σ* and +3.0*σ* levels, respectively. Molecular-graphics representations were produced with *Coot* 1.0 (Emsley *et al*., 2010).

Although H atoms represent a large fraction of the total atomic content of macromolecules (~50% and ~35% of protein and nucleic acid atoms, respectively) their experimental visualization is not straightforward. In X-ray macro-molecular crystallography they contribute little to the total scattering, thus even at (sub-)atomic resolution (<1.2 Å) only a fraction of all H atoms are typically observed in electron density maps (Howard *et al*., 2004; Petrova & Podjarny, 2004). For instance, in the case of the 0.85 Å resolution room-temperature X-ray structure of crambin, less than 50% of all H atoms could be identified (Chen *et al*., 2012). These tend to be the most ordered ones, which are seldom interesting from a functional viewpoint (Fig. 1a). At comparable resolution, H atoms can be expected to be more visible in cryogenic-sample electron microscopy (cryo-EM) maps than in electron density maps due to the nature of the electrostatic potential (Clabbers & Abrahams, 2018; Maki-Yonekura *et al*., 2023). Yamashita *et al*. (2021) analysed H atom density from X-ray crystallo-graphic and cryo-EM single-particle analysis (SPA) data for apoferritin structures deposited in the PDB (Berman *et al*., 2000) and EMDB (Lawson *et al*., 2016), highlighting that even at 2.0 Å resolution it is possible to see some H atoms in cryo-EM maps. For extremely well-behaved samples, the recent ‘resolution revolution’ in cryo-EM SPA has allowed atomic resolution to be achieved (Nakane *et al*., 2020; Yip *et al*., 2020). In the structure of apoferritin at 1.2 Å resolution, most H atoms (approximately 70%) are easily discernible (Fig. 1b). However, a recent microcrystal electron diffraction (microED) experiment on triclinic lysozyme reported at subatomic resolution only allowed the identification of 35% of H atoms (Clabbers *et al*., 2022).

Neutron macromolecular crystallography is a powerful technique that allows the direct visualization of H atoms at more conventional resolutions (Blakeley & Podjarny, 2018). In contrast to X-rays, which interact with atomic electron clouds, neutrons are scattered by nuclei (Fermi & Marshall, 1947). Atoms that are abundant in macromolecules typically possess positive neutron scattering lengths (0.665 × 10^−12^, 0.936 × 10^−12^ and 0.581 × 10^−12^ cm for C, N and O, respectively) that contribute favourably to the signal-to-noise (S/N) ratio of Bragg peaks. Although the scattering length of the common protium isotope (^1^H; note that in this article we use the conventional ^1^H and ^2^H notation to indicate protium and deuterium isotopes, respectively, whilst we use H when referring to hydrogen atoms in general) is small and negative (–0.374 × 10^−12^ cm), its replacement with the heavier deuterium isotope ^2^H (scattering length 0.667 × 10^−12^ cm) makes them readily visible in neutron diffraction maps at 2.0–2.5 Å resolution or better (Fig. 1c). Another important advantage of neutron diffraction for structure determination is the absence of global and specific radiation-induced damage, which can be a serious limitation when using X-ray or electron sources (Baker & Rubinstein, 2010; Garman, 2010).

Crystallographic refinement is one of the final steps in the process of solving a macromolecular structure by diffraction methods (Tronrud, 2004). Various protocols are applied to maximize the agreement between the diffraction data and model parameters, which typically include atomic coordinates, atomic displacement parameters (ADPs) and occupancy values (Shabalin *et al*., 2018). Refinement of macromolecular models using neutron diffraction data can currently be carried out using packages initially developed for X-ray crystallographic refinement and modified to include neutron scattering lengths and the ability to deal with the refinement of individual H atom positions. They include the *nCNS* patch (Adams *et al*., 2009), which is an extension of the *Crystallography and NMR System* (*CNS*) package (Brünger *et al*., 1998), and *SHELXL2013* (Gruene *et al*., 2014). *SHELXL*2013 is the most recent version of the *SHELXL* refinement program originally developed for small molecules and later adapted to macro-molecules (Sheldrick, 2015). Another widely used package for neutron refinement is *phenix.refine* (Afonine *et al*., 2012), which is distributed as a part of the *Phenix* suite (Liebschner *et al*., 2019). This program also includes the option of performing joint neutron/X-ray refinement, a concept first introduced in the field of small-molecule crystallography (Coppens *et al*., 1981) and later applied to macromolecules with its *nCNS* implementation. Although effective joint neutron/X-ray refinement ideally requires the two data sets to be collected from the same crystal under the same conditions, it has the great advantage of increasing the available experimental data, thus compensating for the increased number of parameters arising from the explicit addition of H atoms to the model.

Here, we describe an extension of the crystallographic refinement package *REFMAC*5 (Murshudov *et al*., 2011) from the *CCP*4 suite (Agirre *et al*., 2023) for the refinement of macromolecular models using neutron crystallographic data. Our implementation introduces a new parameter, dubbed the ‘deuterium fraction’, representing the ^1^H/^2^H fraction that is refined during the optimization procedure. It also allows the effective use of stereochemical restraints from high-resolution reference structures, if available. We have tested *REFMAC*5 (version 5.8.0415) for the refinement of neutron models using ^1^H/^2^H fraction parameters for selected or all H atoms together with restraints to a high-resolution known X-ray reference structure. Our evaluation involved the re-refinement of 97 PDB entries and one novel structure (FutA). The results of the refinement process are discussed in this study.

## 2 Methodology and results

### 2.1 Reassessment of *X*—H restraint distances for macromolecular refinement

Macromolecular crystallographic refinement takes advantage of prior chemical knowledge. Information on ‘ideal’ bond lengths, bond angles and other chemical properties are incorporated into the target function and used in restrained refinement as subsidiary conditions to improve the model parameters (Waser, 1963; Diamond, 1971; Jack & Levitt, 1978; Konnert & Hendrickson, 1980). Much of the available prior chemical knowledge used in macromolecular crystallographic refinement derives from high-resolution small-molecule X-ray diffraction experiments and the corresponding structures deposited in databases such as the Cambridge Structural Database (CSD; Groom *et al*., 2016) and the Crystallography Open Database (COD; Gražulis *et al*., 2012). The values of *X*—H (where *X* is a non-H ‘parent’ atom) bond lengths derived from X-ray diffraction experiments reflect the relative positions of the atomic electron clouds. However, the distances between H nuclei and their parent atoms are longer than those between the electron clouds. This is because the valence electron density for H atoms is shifted towards their parent atoms (Coppens, 1997). Thus, to properly model and refine macromolecular models against neutron diffraction data, bond-distance information should take this into account.

In addition to X-ray crystallographic structures, the CSD also contains a limited set of small-molecule structures determined by neutron crystallography. Neutron entries in the CSD have almost doubled in recent years, from 1213 in 2009 to 2362 (1452 organic and 910 metal–organic compounds) in 2021. An analysis of *X*—H bond lengths using the 2009 CSD neutron database was reported by Allen & Bruno (2010) that reassessed information derived from the limited earlier data of the late 1980s and early 1990s (Allen *et al*., 1987, 1992; Orpen *et al*., 1989). We took advantage of the recent enrichment in neutron structures in the CSD and re-evaluated *X*—H bond-length values. We employed the same approach as Allen & Bruno (2010) by selecting nonpolymeric organic compounds without disorder and with *R* factors ≤ 0.075 (647 entries). Entries derived from powder diffraction data were excluded. Neutron entries were retrieved using *ConQuest* (Bruno *et al*., 2002) and mean, median and standard deviation values for the *X*—H bond-length distributions were estimated using *Mercury* (Macrae *et al*., 2020). O—H and N—H bond lengths were estimated by removing groups involved in very short hydrogen bonds, as reported by Allen & Bruno (2010).

In an orthogonal approach, we also derived *X*—H nuclear distances from quantum-mechanics (QM) calculations. Initially, the stereochemical restraints generator *AceDRG* (Long *et al*., 2017) was employed to provide initial coordinates for 2652 molecules constituted of twenty or fewer atoms selected from DrugBank (Wishart *et al*., 2018). The cutoff value on atom numbers was chosen to ensure computational efficiency while providing a pool size comparable to that of CSD entries. For geometry optimization, density functional theory (DFT) calculations were carried out with the self-consistent field wavefunction of restricted Hartree–Fock type as implemented in *GAMESS-US* (Schmidt *et al*., 1993). The hybrid generalized gradient approximation functional, B3LYP, was used with the (6-311++G**) basis set that includes both polarization and diffuse functions. The solvent effect was calculated using the polarizable continuum model with water as solvent. More than 70% of the calculations ran successfully, producing optimized coordinates for 1874 out of 2652 molecules. We did not perform a detailed analysis of calculations that ended prematurely.

**Figure 2:**
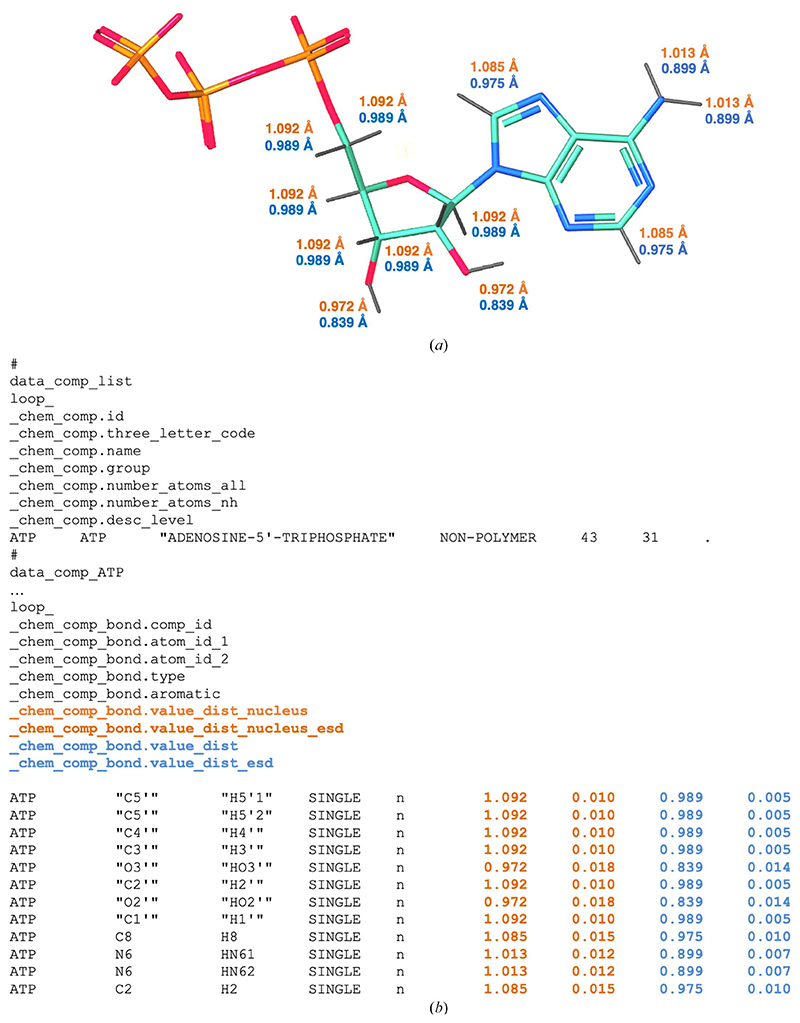
Example of a dictionary mmCIF file from the updated version of *AceDRG*. *(a)* 3D representation of the adenosine triphosphate (ATP) monomer. N, C, O and P atoms are shown in blue, teal, red and orange, respectively. *X*—H bonds are represented by grey sticks with their nuclear and X-ray diffraction-derived bond lengths (in Å) highlighted in orange and light blue, respectively. (*b*) Extract from the monomer description of the ATP component dictionary. The category _chem_comp_bond describes the bonded atoms, bond types and the ideal values of bond lengths and uncertainties associated with them. In this example, we show the ideal *X*—H bond lengths and standard deviations for nucleus positions (_chem_comp_bond.value_dist_nucleus and chem_comp_bond.value_dist_nucleus_esd; orange) and electron positions (_chem_comp_bond.value_dist and _chem_comp_bond.value_dist_esd; light blue).

Table 1 summarizes nuclear bond distances for the most common *X*—H bond classes. It also provides the values as reported by Allen & Bruno (2010) for reference. Overall, the recent nuclear bond distances derived from the CSD in 2021 are fully consistent with those previously derived in 2009. Nuclear distances obtained from theoretical calculations are also consistent with the experimentally derived values. The *AceDRG* data table has been updated to use the median (*m*) and standard deviation (*σ*) values for all *X*—H nuclear distances from the CSD 2021 data (Fig. 2a).

### 2.2 Inclusion of *X—*H nuclear distances in the *CCP*4 Monomer Library (*CCP*4-ML)

The *CCP*4-ML, also referred to as the *REFMAC*5 dictionary (Vagin *et al*., 2004), currently contains close to 35 300 entries for all standard and most nonstandard amino acids, nucleotides, saccharides and various ligands. Each entry, identified as a monomer, possesses a unique code and provides stereochemical information about the constituent atoms, bond distances, bond angles and torsion angles as well as stereo-chemical centres and planes. Statistics for these geometric parameters have been generated by *AceDRG* using data from the COD. In addition, the *CCP*4-ML also contains more than 100 descriptors that specify covalent linkages between monomers and associated chemical modifications. The latter define all of the chemical and geometric changes that occur to monomers following chemical reactions (for example removal of one of the O atoms in peptide-link formation). Covalent links refer to covalent interactions between monomers (for example, peptide links, sugar-peptide links, DNA/RNA links; Nicholls, Joosten *et al*., 2021; Nicholls, Wojdyr *et al*., 2021).

The *CCP*4-ML has recently been updated to contain *X*—H nuclear distances (orange in Fig. 2b) as _chem_comp_ bond.value_dist_nucleus and _chem_comp_bond.value_dist_nucleus_esd in addition to the distances between electron clouds (light blue in Fig. 2b) (Nicholls, Wojdyr *et al*., 2021). *X*—H nuclear distances can now also be used to refine models from electron-derived experiments (cryo-EM SPA and microED), as both H atom ‘positions’ (electron and nucleus) contribute to the scattering.

### 2.3 *CCP*4 implementation of neutron macromolecular crystallographic refinement

#### 2.3.1 ‘Deuterium fraction’ parametrization

Neutron crystallographic experiments on macromolecules are typically carried out on ^1^H/^2^H-exchanged crystals to maximize the S/N ratio (Kossiakoff, 1984). This can be performed by replacing exchangeable ^1^H atoms with ^2^H by soaking macromolecular crystals in deuterated media (Niimura & Podjarny, 2011). Alternatively, perdeuteration, which replaces all H atoms with ^2^H, can be carried out at the protein-production stage by overexpressing the protein(s) of interest in *Escherichia coli* or yeast strains in heavy water-based medium supplied with a perdeuterated carbon source such as glycerol. Protein perdeuteration is a more effective method of improving the S/N ratio as it dramatically lowers the incoherent background while enhancing the coherent scattering signal (Shu *et al*., 2000; Fisher *et al*., 2014). In addition, it avoids map-cancellation issues due to the negative scattering length of protium (Blakeley & Podjarny, 2018; Logan, 2020). Currently, most neutron entries in the PDB (157 out of 213) reflect experiments carried out on partially deuterated samples, as ^1^H/^2^H exchange is simpler and less expensive than perdeuteration. However, the establishment of dedicated deuteration facilities and advanced experimental protocols have made perdeuteration more accessible to users (Meilleur *et al*., 2009; Budayova-Spano *et al*., 2020; Pierce *et al*., 2020).

**Figure 3:**
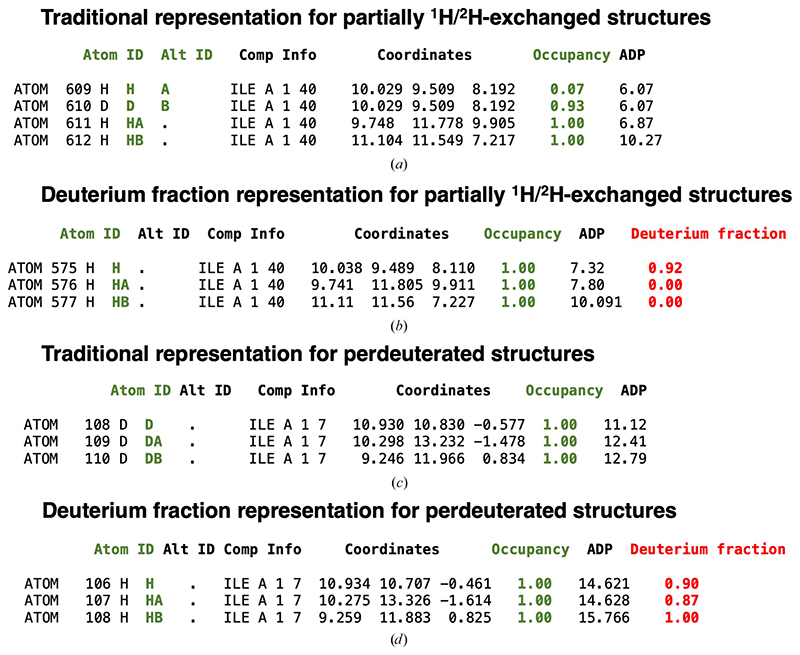
Comparison between the traditional representation of partially ^1^H/^2^H-exchanged structures and perdeuterated structures and the deuterium fraction representation in mmCIF files. (*a*) Traditional exchangeable ^1^H/^2^H sites representation extracted from the mmCIF file of PDB entry 1vcx. The ^1^H atom bonded to the main-chain N atom of Ile40 is partially exchanged with ^2^H. ^1^H and ^2^H isotopes have separate atom rows in the atom table with alternative locations A and B (green). The sum of their total occupancy (green) is set to 1.0 (the occupancy values of the ^1^H and ^2^H atoms are 0.07 and 0.93, respectively). The H atoms bonded to CA and CB of Ile40 are not exchanged during the partial deuteration procedure; their occupancy value is equal to 1.0. (*b*) Deuterium fraction representation created by *REFMAC*5. A new column has been created that specifies the fraction of the deuterium substitution (where 100% is fully deuterated) for the exchanged H atoms. ^2^H atoms are not present in the atom table, only ^1^H atoms with the corresponding deuterium fraction parameters (red). The ^1^H atom bonded to the main-chain N atom of Ile40 has a deuterium fraction value of 0.92, while the ^1^H atoms bonded to CA and CB of Ile40 are not exchanged, hence the deuterium fraction for these H atoms is zero. (*c*) Traditional perdeuterated sites representation extracted from the mmCIF file of PDB entry 3rz6. Here, all of the H atoms of Ile7 have been substituted with ^2^H atoms. There are no ^1^H atoms in the traditional perdeuterated structures. The occupancy of the ^2^H atoms is set to 1.0 by convention. (*d*) Deuterium fraction representation for perdeuterated structures. All ^2^H atoms are converted to ^1^H atoms, the corresponding deuterium fractions are refined and values close to 1.0 are obtained.

In the refinement procedure implemented in *REFMAC*5, we have introduced a new quantity that represents the deuterium fraction for individual H atoms. This method is similar to the ‘deuterium saturation’ implemented in *SHELXL* (Gruene *et al*., 2014). In this parametrization, protium ^1^H and deuterium ^2^H isotopes at each H position are not considered as separate entities. Instead, H atoms are represented by a unique set of coordinates that are associated with their isotope fraction, which is optimized during the minimization of the target function. The scattering factor for the ^1^H/^2^H mixture is calculated using

(1) $$f_{i}(s)=\left(1-m_{i}\right)b_{\text{H}}+m_{i}b_{\text{D}},$$

where *f_i_*(*s*) is the total contribution of protium and deuterium isotopes to the scattering factor of the *i*th H atom, *s* is the Fourier space vector, *m_i_* is the deuterium fraction parameter, which is an adjustable parameter, and *b*_H_ and *b*_D_ are the neutron scattering lengths of the ^1^H and ^2^H isotopes, respectively. Neutron scattering lengths are tabulated in the *CCP*4 atomsf_neutron library, retrieved from https://www.ncnr.nist.gov/resources/n-lengths/list.html (Sears, 1992). The refined output model in mmCIF format contains only H atoms (no ^1^H/^2^H or ^2^H sites) and a new _atom_site.ccp4_deuterium_fraction column representing the value of the deuterium fraction for each of the H atoms in the model. Users have the option to refine deuterium fraction parameters for either only polar or all H atoms. This method simplifies the model output as there is no ^1^H/^2^H duplication for the same set of coordinates, for example, when alternative conformations are introduced into the structure (Figs. 3a and 3b). The presence of only ‘generalized’ H atoms with their corresponding deuterium fraction parameter also reduces the risk of bookkeeping errors. In the deuterium fraction representation, all ^2^H atoms are converted to H atoms and their presence is indicated by their corresponding deuterium fractions (Figs. 3c and 3d). We note that this new item can only be added to mmCIF files, which is now the model deposition standard. For PDB files that have fixed-column format, ^1^H and ^2^H are present at each H position and the deuterium fraction is indicated in the occupancy column.

#### 2.3.2 Reference structure restraints

Neutron macro-molecular crystallographic data often suffer from limited completeness and high resolution is not always achievable. Therefore, a useful strategy to increase the data-to-parameter ratio in refinement is that of joint neutron/X-ray refinement, provided that an isomorphous X-ray data set is available. This approach, which was originally implemented in *nCNS* and is available within *phenix.refine* in the *Phenix* suite, has been employed for the refinement of several macromolecular structures (Liebschner *et al*., 2018).

Neutron diffraction data sets are often of poorer quality compared with X-ray data. The low flux of available neutron beams requires either large crystals or very long exposure times for smaller crystals to obtain measurable diffraction data. Consequently, neutron data sets often have low completeness due to the limited data-collection time available on neutron crystallographic instruments. Additionally, the incoherent scattering of H atoms can lead to low S/N ratios.

Combining two sources of information, X-ray and neutron, can potentially mitigate some of the challenges when refining models against neutron data alone. The current joint refinement method uses a combined target function to optimize a single atomic model simultaneously against two data sets (X-ray and neutron; Afonine *et al*., 2010; Liebschner *et al*., 2020). To satisfy the refinement of the target function, the X-ray and neutron crystals should be isomorphous and ideally the data should be collected under the same conditions. This, however, cannot always be accomplished.

Any differences in the underlying structures of macro-molecules analysed using different experimental methods can cause problems and require special consideration. This has been observed, for example, in the joint refinement of macromolecular models against X-ray and NMR data (Kovalevskiy *et al*., 2018). Joint refinement can be useful in identifying discrepancies between structures obtained under different experimental conditions. However, if attempting to achieve a single model, it is important to ensure that any approach involving the co-utilization of data from different experimental sources does not suffer from excessive bias due to fundamental structural differences. Therefore, there is a preference to avoid joint refinement in cases where other strategies to stabilize neutron refinement exist.

One such strategy is to utilize structural information from homologous X-ray models via the use of external restraints. Such restraints have been useful in the refinement of low-resolution X-ray (Headd *et al*., 2012; Nicholls *et al*., 2012; Smart *et al*., 2012; Schröder *et al*., 2014; Sheldrick, 2015; van Beusekom *et al*., 2018) and cryo-EM (Afonine *et al*., 2018; Nicholls *et al*., 2018) structures. This approach is robust to structural differences between the target and reference models by employing an anharmonic penalty function, which avoids pulling the model into conformations that are not supported by the data.

The purpose of external restraints is twofold. Firstly, to inject prior structural information: the target (neutron) model is pulled towards the conformation adopted by the reference (X-ray) structure, which helps to improve the model stereochemistry/geometry. Secondly, to increase the effective data-to-parameter ratio, thus stabilizing refinement and helping to avoid overfitting. The importance of the latter should not be underappreciated, especially given that neutron data are typically limited and noisy. This approach can be applied if a high-resolution model related to the target structure to be refined is available. Fortunately, when performing neutron crystallographic studies of macromolecules, the corresponding high-resolution X-ray models are invariably determined first and thus are generally available. Given that X-ray models provide significantly more accurate coordinates for all non-H atoms than their neutron counterparts, their use as a source of prior structural information appears to be a reasonable approach towards improving neutron refinement.

The *CCP*4 program *ProSMART* (Nicholls *et al*., 2014) generates such external restraints by distilling the local structure of a known reference model. Here, we used *Pro-SMART* to identify matching atoms by aligning the target model and an X-ray reference model before generating interatomic distance restraints between proximal non-H atoms within a given distance threshold (default 4.2 Å), which should be long enough to capture information about secondary structure whilst being short enough to allow differences in global conformation. The resulting external restraints were subsequently used by *REFMAC*5 during refinement of the target neutron model.

### 2.4 Performance analysis by re-refinement of PDB entries

To test our current implementation, we re-refined 97 of the available neutron PDB entries (45.5% of the total) using *REFMAC*5. Of these, 55 are structures that were originally refined against neutron data only and 42 are entries deposited following a joint neutron/X-ray refinement protocol. We selected our test pool based on the availability of experimental data (including complete cross-validation sets) and a wide resolution range (upper limit 1.05–2.75 Å).

For each entry, coordinate files (in PDB and mmCIF format) and crystallographic data (mmCIF format) were downloaded from the PDB. Each mmCIF reflection file was then converted into MTZ format, which serves as the standard format used by *CCP*4 programs (Agirre *et al*., 2023). For the ‘neutron-only’ entries, the *CCP*4 program *CIF*2*MTZ* was utilized to convert mmCIF to MTZ format. For entries refined using a joint X-ray/neutron protocol, their mmCIF reflection files should contain two distinct data blocks: one for X-ray diffraction and one for neutron diffraction. However, a few entries have been erroneously deposited with a single data set. Since the refinement process within *REFMAC*5 was only performed against neutron reflections, those were extracted and converted to MTZ format using *GEMMI* (Wojdyr, 2022). In cases where only intensities were available, they were converted into amplitudes using the *Servalcat* ‘fw’ function (Yamashita *et al*., 2021), which implements the French–Wilson procedure (French & Wilson, 1978).

To compare refinement statistics with those reported in the PDB, all ^1^H and ^2^H atoms present in the models were retained without regeneration. *REFMAC*5 is able to read ^1^H/^2^H sites and ^2^H atoms using the *Servalcat REFMAC*5 controller (‘refmacat’), which uses *GEMMI* for restraint generation (Yamashita *et al*., 2023). ^2^H atoms are converted to H atoms with deuterium fraction parameters by *GEMMI*, and their distances are adjusted using nuclear values from the *CCP*4-ML. In cases such as PDB entry 5ksc, where the original model does not contain any ^1^H (or ^2^H) atoms except for water molecules, *GEMMI* was employed to add them at riding positions.

If H atoms are generated, it is necessary to initialize their deuterium fraction prior to refinement. Users can choose to initialize all H atoms or only polar H atoms. For perdeuterated structures, in which all H atoms are replaced by ^2^H atoms, the initialization process sets the deuterium fraction parameter to 1 for all H atoms. In the case of ^1^H/^2^H-exchanged structures, the deuterium fraction is only set to 1 for H atoms exchanged with ^2^H. Subsequently, the refinement process is performed to optimize the deuterium fraction. Initialization was not used for the refinement of most of the entries containing ^1^H/^2^H or ^2^H sites, while it was necessary for a few entries, such as PDB entries 1c57, 1cq2, 5ksc and 1xqn, where only ^1^H atoms were present in the models.

Our standard refinement protocol consisted of five cycles of restrained positional and individual ADP refinement using the data in the published resolution range. Three cycles of deuterium fraction refinement were performed after each cycle of individual atomic refinement. For perdeuterated samples we allowed refinement of the deuterium fraction for all H atoms, whilst for ^1^H/^2^H-exchanged samples only polar H atoms had this parameter included in the optimization. H atom positions have been refined individually with all available restraints (bond lengths, angles, planarity and torsion angles) to ensure proper geometry. We found that this procedure allows deuterium fraction parameters to converge as the models had previously been refined by the original depositors.

#### 2.4.1 Re-refinement of PDB entries originally refined against neutron data only

Using the protocol described earlier, we used *REFMAC*5 to re-refine 55 PDB entries that were originally refined using neutron data only. Entries were chosen over a wide resolution range from medium–low resolution (2.7 Å, PDB entry 2efa) to subatomic resolution (1.05 Å, PDB entry 4ar3). *R*-factor statistics for all 55 re-refined models are given in Table 2.

For some entries (for example PDB entries 1wq2, 3rz6, 4c3q, 4fc1, 5a90, 5gx9 and 7kkw in Table 2), we observe that the initial *R*_work_ and *R*_free_ values are higher than those reported in the PDB. In the case of PDB entry 1wq2, the PDB header reports values of 22.9% and 28.9% for *R*_work_ and *R*_free_, respectively, while the paper indicates values of 28.2% and 30.1% (Chatake *et al*., 2003). The latter values are similar to the initial *R* factors from *REFMAC*5 (28.6% and 32.1% for *R*_work_ and *R*_free_, respectively). Following refinement, the *R*_work_ and *R*_free_ values from *REFMAC*5 become comparable to the deposited values, suggesting convergence of the refinement procedure (Table 2).

For several structures, including the low-resolution PDB entries 1c57, 1wq2, 1xqn, 2gve and 2yz4, the medium-resolution PDB entries 3fhp, 3u2j, 4bd1 and 6h1m and the high-resolution PDB entries 2zoi, 2zwb, 3a1r, 4ar3, 4ar4, 4fc1 and 4q49, the *R*_work_ and *R*_free_ values obtained from *REFMAC*5 are lower compared with the deposited values, often improving by ~2–3 percentage points. However, for a few other entries the final *R*-factor values obtained from *REFMAC*5 are slightly higher. One explanation is that in this study the models have been re-refined without any additional refinement strategy that could significantly improve the refinement statistics. For example, the application of TLS refinement (Winn *et al*., 2001, 2003), as well as the use of anisotropic ADP refinement for high-resolution structures and jelly-body restraints, could potentially improve the refined model. One general point of consideration, however, is that the calculation of scaling factors used in the *R*-factor equation is different among refinement packages and this can lead to differences in *R* factors. Although overall *R* values are not the only metrics to consider when evaluating the quality of a structural model, which cannot be properly assessed without careful map analysis, the values obtained from this test set indicate that our implementation for neutron crystallographic refinement performs satisfactorily.

#### 2.4.2 Re-refinement of PDB entries originally refined using a joint neutron/X-ray strategy

We also tested the refinement of 42 models previously obtained through joint neutron/X-ray refinement utilizing solely neutron data and incorporating the deuterium fraction parameterization. Table 3 presents all joint neutron/X-ray models featuring neutron data from lowest to highest resolution that were selected for re-refinement within *REFMAC*5. The table compares the *R*-factor statistics published for these selected entries with the *R* factors obtained through their re-refinement using *REFMAC*5.

The *R*_work_ and *R*_free_ values obtained from *REFMAC*5 [Table 3; Final *R* values (work/free) column] by refining joint models using only neutron data were found to be similar to those obtained from joint neutron/X-ray refinement [Table 3; Published *R* values (work/free) column]. For some entries, the *R* factors are slightly improved compared with the published values. It is widely acknowledged that refinement solely using neutron data may lead to overfitting due to the explicit refinement of H atom parameters. However, the gap observed between the *R*_work_ and *R*_free_ values obtained from *REFMAC*5 is not substantial (the mean Δ*R* is ~6%). Thus, this strategy can be a viable alternative when joint refinement is not feasible.

#### 2.4.3 Re-refinement using external restraints

To improve the quality of neutron atomic models, especially at low resolution, re-refinement was performed by incorporating X-ray reference structure restraints. A subset of models obtained by neutron refinement only and by joint neutron/X-ray refinement, featuring neutron data at low resolution and a few at high resolution, were selected for this analysis. For the ‘neutron-only’ entries (PDB entries 1c57, 2efa, 2yz4 and 2zpp), the corresponding X-ray reference structures were chosen from the PDB based on their high structural similarity to the neutron refined structures. The ‘Find Similar Assemblies’ option in the PDB uses *Structure Similarity Search* (Guzenko *et al*., 2020) to assess global 3D shape similarity, providing a Structure Match Score indicating the probability as a percentage that the structure match is similar to the query. The X-ray structures chosen reported the highest Structure Match Score. If a suitable X-ray reference model is not known, we recommend running a *BLAST* search (Altschul *et al*., 1997) over the whole PDB by inputting the FASTA sequence of the target neutron model.

For the joint neutron/X-ray structures selected a different protocol was applied. Firstly, these models were subjected to refinement against their corresponding X-ray data using *REFMAC*5, with a total of ten refinement cycles. The output model obtained from this refinement process was subsequently employed as a reference model.

*ProSMART* (Nicholls *et al*., 2014) takes as input the neutron target model and X-ray reference structure model in PDB or mmCIF format and generates interatomic distance restraints between proximal non-H atoms reported in a restraint file. The refinement was performed by simultaneously refining non-H atoms of the model by using restraints generated by *ProSMART* and by using the deuterium fraction parametrization for H atoms (twenty refinement cycles inter-leaved with three deuterium fraction refinements^1^). PDB information for the neutron and X-ray models selected, as well as the published refinement statistics and those obtained by *REFMAC*5, are shown in Table 4.

The incorporation of external restraints has been observed to improve both the *R*_work_ and *R*_free_ values for low-resolution neutron structures. Specifically, the *R* factors are improved by ~2–3 percentage points in certain cases (PDB entries 1c57, 2efa, 2yz4 and 4cvi; Table 4, Neutron refinement with external restraints) compared with both the published values and those obtained using deuterium fraction refinement only. Moreover, certain high-resolution structures (PDB entries 3x2o and 3x2p) also demonstrate improved *R* factors, which indicate that these restraints can improve the quality of neutron models regardless of the resolution.

### 2.5 Selected examples of neutron crystallographic refinement

#### 2.5.1 Re-refinement of the neutron structure of chloride-free urate oxidase in complex with its inhibitor 8-azaxanthine

Our re-refinement runs reported in previous sections mainly looked at global refinement statistics. As a selected example that involved a more detailed inspection of neutron maps, we carried out a re-refinement of the joint neutron/X-ray structure of perdeuterated urate oxidase (UOX) in complex with its 8-azaxanthine (8AZA) inhibitor (PDB entry 7a0l; McGregor *et al*., 2021).

In many organisms, the degradation of uric acid (UA) to 5-hydroxyisourate (5-HIU) is catalysed by cofactor-independent UOX (Kahn *et al*., 1997). In a two-step reaction, UA first reacts with O_2_ to yield dehydroisourate (DHU) via a 5-peroxoisourate intermediate (Bui *et al*., 2014). This is then followed by a hydration step, in which DHU is hydroxylated to 5-HIU (Kahn, 1999; Wei *et al*., 2017). The joint structure of perdeuterated UOX in complex with its 8AZA inhibitor, relevant to the hydration step, has recently been determined using X-ray and neutron data at 1.33 and 2.10 Å resolution, respectively (McGregor *et al*., 2021). Joint refinement was carried out with *phenix.refine* (Afonine *et al*., 2010). It showed that the catalytic water molecule (W1) is present in the peroxo hole as neutral H_2_O (D_2_O), oriented at 45° with respect to the organic ligand. It is stabilized by Thr57 and Asn254 on different UOX protomers as well as by an O—H…*π* interaction with 8AZA. The active site Lys10–Thr57 dyad features a charged ${\text{Lys10–NH}}_{3}^{+}$ side chain engaged in a strong hydrogen bond with Thr57^OG1^, while the Thr57^OG1–HG1^ bond is oriented toward the *π* system of the ligand, on average.

Re-refinement of the UOX:8AZA complex with *REFMAC*5 was performed against neutron data alone using deuterium fraction parameterization and external restraints. ^1^H and ^2^H atoms on previously modelled residues and water molecules were maintained at their positions and were not regenerated. Deuterium fraction parameters were refined for all H atoms. H atom positions were refined individually. External restraints were generated using *ProSMART* by first re-refining the model against its X-ray data (ten cycles) and using the output model as a reference structure. Data-collection and refinement statistics are given in Supplementary Table S1.

**Figure 4:**
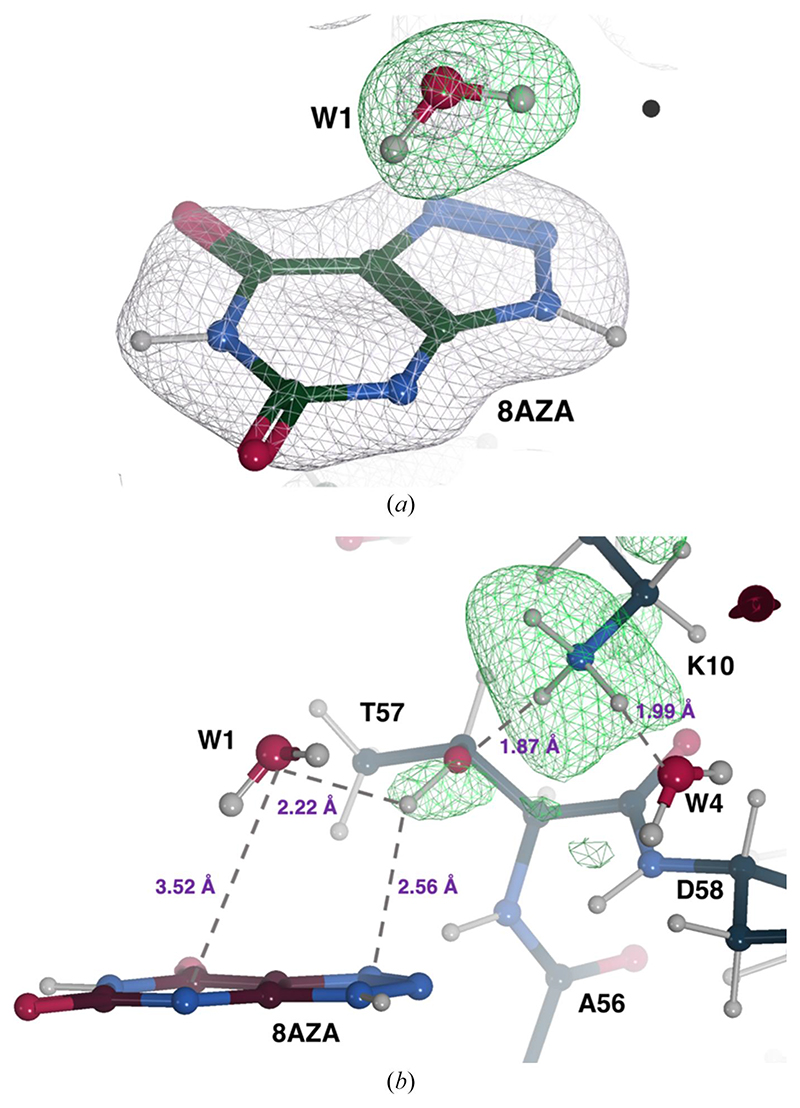
Neutron structure of the UOX:8AZA complex. (*a*) W1 is present as a neutral H_2_O molecule. A 2*mF*_o_ — *DF_c_* neutron scattering length density map for 8AZA and the O atom of W1 is shown in grey at the +1.0*σ* level. An omit *mF_o_ — DF_c_* neutron map indicates the presence of two deuterons (H1 and H2) as suggested by the elongated positive density (in green at the +3.0*σ* level) next to the O atom. (*b*) Representation of a portion of the active site highlighting the protonation of the Lys10–Thr57 dyad and the hydrogen-bonding network. H difference neutron density for Lys10^NZ^ and Thr57^OG1^ is shown in green at the +3.0*σ* and +2.5*σ* levels, respectively. Hydrogen bonds are shown as grey dashed lines and their distances are shown in purple.

8AZA is bound as a monoanion deprotonated at N3 and omit neutron maps confirm that W1 is neutral (Fig. 4a). This is supported by the presence of positive peaks for two ^2^H atoms whose deuterium fraction values refine to 0.77 (H1) and 0.84 (H2). The protonation state of the Lys10–Thr57 active site dyad has also been investigated. Omit neutron maps for Lys10 show that the residue is positively charged due to the presence of a ‘tri-lobe’ density distribution around NZ (Fig. 4b). All H atoms bound to Lys10 refine with a high deuterium fraction parameter value (>0.80). The direction of the OG1–HG1 bond in Thr57 was not easily identified in the original work (McGregor *et al*., 2021). Here, omit maps reveal positive density for Thr57^HG1^ at the 2.5*σ* level (Fig. 4b). We refined the orientation of the OG1–HG1 bond using the *REFMAC*5 ‘hydrogen refine rpolar’ (rotatable polar) option, resulting in an optimal fit to the density. The deuterium fraction parameter for HG1 refined to 0.81. The orientation of the OG1–HG1 bond suggests the formation of another O—H…*π* interaction with N7 of 8AZA at 2.56 Å and a hydrogen bond is also formed between Lys10^HZ1^ and Thr57^OG1^ at a distance of 1.87 Å (Fig. 4b). Overall, our results are fully consistent with those from the previous study (McGregor *et al*., 2021), and mechanistic considerations can be found therein.

#### 2.5.2 Refinement of the neutron structure of the *Prochlorococcus* iron-binding protein FutA

Finally, we employed *REFMAC*5 for the refinement of a novel neutron structure. The marine cyanobacterium *Prochlorococcus* plays a significant role in global photosynthesis (Huston & Wolverton, 2009). However, its growth and productivity are constrained by the limited availability of iron. *Prochlorococcus* encodes the FutA protein that can accommodate the binding of iron in either its ferric (Fe^3+^) or ferrous (Fe^2+^) state. The structure of FutA has recently been determined using a combination of structural biology techniques at room temperature, revealing the redox switch that allows the binding of both iron oxidation states (Bolton *et al*., 2023).

**Figure 5:**
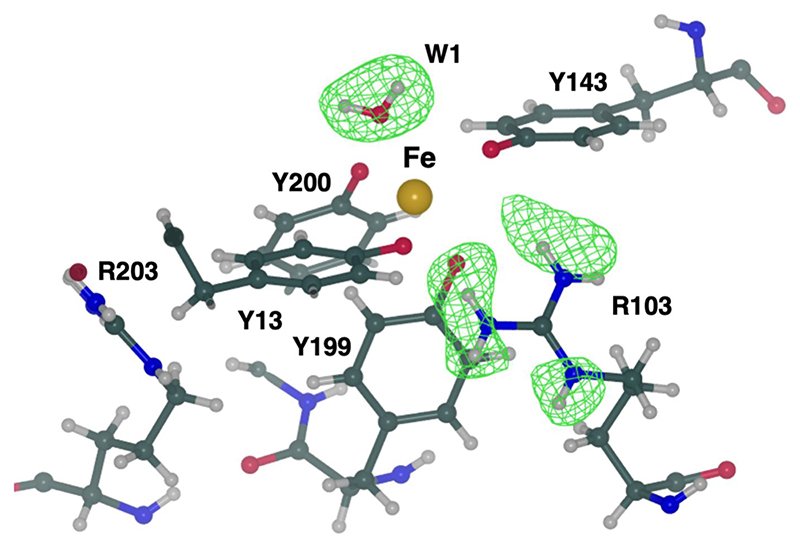
FutA (ferric state) determined by neutron diffraction at 2.1 Å resolution. The iron-binding site is formed by four tyrosines (Tyr13, Tyr143, Tyr199 and Tyr200), a solvent molecule (W1) and Arg103 in the second coordination shell. The positive density (green mesh, *mF_o_ — DF_c_* omit map at the +3.0*σ* level) indicates that these atoms have undergone ^1^H/^2^H exchange and suggests that Arg103 is positively charged whilst W1 is neutral. The side chain of Arg203 is not oriented towards the binding site and does not engage in polar interactions. N, C and O atoms are shown in blue, dark green and red, respectively. Iron is shown in gold and H atoms are in grey.

The X-ray structure of FutA, determined at a resolution of 1.7 Å, shows that the iron-binding site involves four tyrosine side chains (Tyr13, Tyr143, Tyr199 and Tyr200) and a solvent molecule, forming a trigonal bipyramidal coordination. The presence of Arg203 in the second coordination shell suggested the possibility of X-ray-induced photoreduction of the iron centre, leading to a ferrous (Fe^2+^) binding state. To investigate the protonation of active site residues surrounding the iron, the neutron structure of FutA was determined at 2.1 Å resolution using ^1^H/^2^H-exchanged crystals, taking advantage of deuterium fraction refinement (Bolton *et al*., 2023). The final model is characterized by *R*_work_ and *R*_free_ values of 18.2% and 25.0%, respectively. Data-collection and refinement statistics are given in Supplementary Table S2. The neutron structure reveals that the side chain of Arg103 is protonated and thus carries a positive charge, with all of its exchangeable H atoms refining with deuterium fraction parameter values of >0.50 (Fig. 5). Neutron maps suggest that the iron-coordinating residues Tyr13, Tyr143, Tyr199 and Tyr200 exist as tyrosinates. The H-omit map for the water molecule (W1) confirms its presence as neutral H_2_O, supported by the presence of positive peaks for two H atoms whose deuterium fraction values refine to 0.84 (H1) and 1.0 (H2) (Fig. 5). In contrast to the room-temperature X-ray structure, Arg203 is not involved in any interactions and does not contribute to the second coordination shell. Consequently, the iron-binding site is composed of four negatively charged tyrosinates, a positively charged arginine in the second shell and a neutral water (W1), suggesting that this coordination cages neutralized ferric iron. This was further confirmed by the serial femtosecond X-ray structure and electron paramagnetic resonance (EPR) measurements. Coordinates and structure factors of the neutron FutA structure have been deposited in the PDB as entry 8oen. This represents the first neutron structure to be refined using *REFMAC*5 and deposited within the PDB. Further mechanistic information on FutA is discussed in a separate publication (Bolton *et al*., 2023).

## 3 Conclusions and availability

Neutron crystallography offers a unique advantage in the determination of H atom positions, enabling the investigation of many biological processes. Despite its great potential in structural biology, the number of biological structures deposited in the PDB to date (25 July 2023) using neutron-only data (or joint neutron/X-ray) is extremely small (213) compared with those solved by X-ray crystallography (176 935), nuclear magnetic resonance (NMR; 14 034) and electron microscopy (EM; 16 239). This is due to technical limitations such as low neutron beam flux, long data-collection times and limited access to neutron beamlines. Nonetheless, recent advances in instrumentation, experimental protocols and computational tools have significantly advanced the field. As a result, the number of neutron structures deposited in the PDB has significantly increased in the last few years. The period 2015–2022 alone has seen the deposition of more than half of the total neutron structures (130 out of 213) and this is likely to further accelerate in the coming years.

The *CCP*4 suite now provides tools for the refinement of macromolecular models using neutron diffraction data. Recent developments include the extension of the *CCP*4 Monomer Library by incorporating H atom nucleus distances. These restraints are required to ensure the correct H atom positions in neutron crystallographic refinement. Moreover, the inclusion of H nucleus positions holds potential for the further refinement of H atoms of cryo-EM models, as both H atom positions (electron and nucleus) contribute to the scattering. New features and refinement strategies have been implemented in *REFMAC*5 for the refinement of neutron models: specifically, the introduction of the deuterium fraction parameter for H atoms. One of the benefits of this approach is that it generates models containing only H atoms, without any ^1^H/^2^H or ^2^H sites. For each H atom, the models incorporate a deuterium fraction parameter that indicates the level of deuteration in the sample. This results in clearer and more easily interpretable models, minimizing the bookkeeping errors that may arise when alternative conformations are present in the models. Re-refinement of neutron structures using *REFMAC*5 has yielded *R*-factor values that are in line with the originally deposited values, including those obtained previously through joint neutron/X-ray techniques. Additionally, for certain neutron entries the refinement process has led to improvements in model quality. Another valuable strategy is the use of external reference structure restraints during the refinement of models obtained by neutron diffraction. Incorporating restraints from X-ray reference structures has demonstrated an enhancement in the accuracy and reliability of neutron models, particularly in low-resolution cases.

The ability to perform neutron crystallographic refinement using *REFMAC*5 will be available in *CCP*4*i*2 (Potterton *et al*., 2018) and *CCP*4 Cloud (Krissinel *et al*., 2022) in an upcoming version of *CCP*4 that uses *Refmacat* instead of *REFMAC*5. This option can be enabled by selecting the appropriate diffraction experiment type (X-ray, Electron or Neutron) in the ‘Advanced’ tab of the Refinement task interface, in which case the appropriate form factors and relevant default behaviours are used during model refinement. In Neutron mode, the graphical interface provides the ability to choose whether to refine all, only polar or only rotatable polar H atom positions, to use H atom torsion-angle restraints, to refine the ^1^H/^2^H fraction for all H atoms (for perdeuterated crystals) or just polar H atoms (for ^1^H/^2^H exchanged samples), and the choice of whether to reinitialize ^1^H/^2^H fractions prior to refinement. Relevant keywords and documentation for neutron crystallographic refinement will be available in the documentation section of the *CCP*4 website (https://www.ccp4.ac.uk/).

## Supplementary Material

**Supporting information:** this article has supporting information at journals.iucr.org/d

Supplementary Tables

## Figures and Tables

**Table 1:** Nuclear *X*—H bond lengths. *X* represents (C, N, O, S) atoms covalently bound to H. For C, different types of hybridization are given, with C(ar) indicating aromaticity. The first set of values (CSD-2009) is that of Allen & Bruno (2010) obtained from an analysis of curated neutron diffraction structures available within the CSD in 2009. The values in the CSD-2021 column are updated values from our analysis using curated CSD entries as of 2021. The values in the QM column are the result of QM calculations carried out as described in the text. The letters *μ, σ*, *m* and *n* represent the mean, standard deviation, median and number of observations, respectively, for each *X*—H class. For QM, the standard deviation is not applicable. All bond lengths are in Å.

	CSD-2009				CSD-2021				QM			
	*μ*	*σ*	*m*	*n*	*μ*	*σ*	*m*	*n*	*μ*	*σ*	*m*	*n*
C(*sp*^1^)—H	1.042	0.022	1.044	5	1.042	0.022	1.044	5	1.063	—	1.063	9
C(*sp*^2^)—H	1.082	0.013	1.084	109	1.083	0.015	1.085	163	1.087	—	1.085	538
C(*sp*^3^)—H	1.089	0.010	1.091	1118	1.087	0.010	1.092	1397	1.093	—	1.093	12985
C(ar)—H	1.083	0.017	1.085	721	1.084	0.018	1.085	1251	1.083	—	1.083	3906
C(*sp*^2^)—N—H_2_	1.013	0.010	1.012	141	1.014	0.012	1.013	177	1.010	—	1.009	1055
C(*sp*^3^)—N—H_2_	1.002	0.010	1.002	4	1.002	0.052	1.018	68	1.020	—	1.019	1172
C(*sp*^3^)—O—H	0.970	0.012	0.971	169	0.969	0.018	0.972	186	0.966	—	0.964	1229
S—H	1.338	—	1.338	1	1.338	—	1.338	1	1.345	—	1.345	83

**Table 2:** Re-refinement of selected neutron-only models from the PDB. Comparison of published *R*-factor statistics and those obtained by re-refinement using *REFMAC*5.

PDB information			*REFMAC*5 refinement statistics			
PDB code	Published resolution range (low–high) (Å)	Published *R* values (work/free) (%)	Initial *R* values (work/free) (%)	Final *R* values (work/free) (%)	Data completeness (%)	No. of reflections
1c57	15.79–2.40	27.0/30.1	29.7/33.0	19.9/25.4	87.38	8129
1cq2	6.00–2.00	16.0/25.0	18.6/25.7	14.9/24.7	91.07	7528
1iu6	10.00–1.60	20.1/22.8	21.1/23.4	18.4/22.9	87.16	5775
1v9g	25.05–1.80	22.2/29.4	26.9/33.5	22.9/33.7	85.31	1949
1vcx	27.60–1.50	18.6/21.7	19.0/21.7	17.8/21.8	81.94	6620
1wq2	20.00–2.40	22.9/28.9	28.6/32.1	21.5/28.7	92.29	6232
1xqn	32.82–2.50	26.6/32.0	30.0/31.5	23.0/29.4	74.99	6088
2dxm	8.00–2.10	19.7/26.0	20.2/26.6	18.8/26.4	64.12	20178
2efa	80.00–2.70	21.6/29.1	24.3/27.6	22.3/28.0	95.66	2154
2gve	10.00–2.20	26.8/31.9	26.3/30.3	23.4/29.8	93.73	22133
2wyx	19.41–2.10	22.3/25.8	25.8/28.7	22.5/27.7	86.85	15033
2xqz	53.19–2.10	22.5/25.9	25.9/29.1	19.0/26.5	77.26	13374
2yz4	33.64–2.20	27.9/31.2	28.1/31.3	22.0/27.6	66.06	8044
2zoi	70.00–1.50	19.2/21.9	19.9/22.4	18.5/21.5	89.64	14526
2zpp	20.00–2.50	22.1/26.0	22.8/26.8	20.5/25.9	98.61	2612
2zwb	20.00–1.80	22.3/24.7	22.8/25.0	18.0/23.1	95.93	9862
3a1r	30.84–1.70	19.5/23.8	18.5/22.9	16.1/21.8	81.89	10300
3fhp	41.43–2.00	16.8/24.7	18.6/23.4	16.8/22.2	81.46	4615
3kmf	20.00–2.00	25.0/30.0	28.9/29.1	27.9/31.0	86.35	31611
3q3l	36.42–2.50	22.1/26.8	23.0/26.8	22.9/26.6	73.25	27157
3ryg	27.11–1.75	18.1/20.0	22.8/23.8	20.4/25.5	92.21	4884
3rz6	21.77–1.75	20.8/23.8	25.2/25.6	21.3/25.1	79.55	4215
3rzt	27.21–1.75	20.2/24.9	25.4/29.3	21.9/29.2	75.60	3989
3ss2	24.33–1.75	21.1/24.2	23.9/26.2	20.1/26.0	77.19	4080
3u2j	12.10–2.00	23.2/27.2	23.5/27.0	22.4/26.5	86.54	14521
4ar3	15.71–1.05	19.9/23.7	19.7/23.0	18.8/22.4	88.73	21580
4ar4	27.46–1.38	18.6/22.6	17.9/22.1	15.5/21.0	91.56	9958
4bd1	9.97–2.00	21.9/25.7	22.4/25.0	14.6/17.6	87.72	18290
4c3q	10.00–2.20	19.2/24.0	21.3/25.1	17.3/24.2	88.19	13256
4fc1	10.00–1.10	21.1/25.3	22.8/25.5	21.5/24.1	76.85	10549
4g0c	20.00–2.00	26.7/28.3	27.2/26.0	24.3/28.1	84.40	13742
4k9f	27.14–1.75	19.9/24.1	21.6/26.0	17.8/25.7	74.37	3671
4q49	20.00–1.80	18.7/21.5	18.6/20.9	15.5/20.4	89.45	18803
4y0j	20.00–2.00	26.3/29.1	29.2/30.6	26.6/31.8	80.89	13095
4zz4	51.19–1.80	19.7/22.1	19.5/20.6	17.7/23.4	71.33	7524
5a90	38.91–1.70	19.2/22.7	21.1/24.1	18.9/23.2	88.61	28772
5gx9	33.45–1.49	15.8/20.0	17.3/21.0	17.0/20.9	93.50	14375
5ksc	10.00–2.10	24.0/28.0	29.6/31.4	23.5/30.9	70.78	12424
5mnx	22.17–1.42	16.6/20.6	18.5/21.9	18.3/21.8	90.57	36197
5mny	22.09–1.43	16.4/19.3	18.1/20.5	18.2/20.2	93.83	36397
5mnz	21.32–1.45	16.9/20.1	18.1/21.1	18.0/21.0	90.20	33722
5mo1	19.68–1.49	17.5/21.6	18.6/22.3	18.9/22.5	93.38	32293
5mo2	22.14–1.50	16.1/20.0	17.4/20.8	17.2/20.5	88.77	30027
5ty5	14.78–2.30	23.9/25.2	27.2/27.0	24.4/29.8	73.88	34761
5vg1	12.00–2.10	18.7/26.5	21.4/27.5	19.8/27.0	75.47	13304
5vnq	16.70–2.20	24.2/28.0	27.5/30.1	23.7/29.0	71.29	7734
5zo0	22.86–1.65	18.6/22.9	19.7/23.2	18.2/22.4	86.21	21422
6c78	14.76–1.75	18.9/21.7	23.9/25.6	19.8/24.9	85.31	25773
6gtj	32.66–1.80	23.2/27.6	24.7/28.6	19.7/27.6	78.58	21858
6h1m	21.76–2.15	21.8/24.9	22.3/25.3	18.5/22.5	91.42	12707
6l26	24.76–1.44	16.8/20.6	18.9/22.1	16.9/21.0	88.73	33763
7jor	17.23–2.05	24.9/28.8	25.4/29.5	23.2/29.5	79.44	12714
7kks	14.64–2.20	25.7/28.2	28.1/30.7	22.4/29.0	98.34	23326
7kkw	14.65–2.30	24.9/30.2	27.9/31.8	22.5/31.6	98.38	20628
7vei	17.70–2.00	17.0/21.5	16.8/21.5	14.2/21.3	98.30	7555

**Table 3:** Re-refinement of selected joint neutron/X-ray models from the PDB using neutron diffraction data only. Comparison of published *R*-factor statistics and those obtained by re-refinement using *REFMAC*5.

PDB information			*REFMAC*5 refinement statistics			
PDB code	Published resolution range (low–high) (Å)	Published *R* values (work/free) (%)	Initial *R* values (work/free) (%)	Final *R* values (work/free) (%)	Data completeness (%)	No. of reflections
2r24	40.11–2.19	25.7/29.1	25.9/29.7	22.1/30.0	72.76	10892
3r98	53.54–2.40	20.7/25.1	20.8/26.1	18.4/25.7	74.99	11610
3r99	53.54–2.40	20.7/25.0	20.8/26.1	18.5/25.6	74.99	11610
3vxf	44.87–2.75	18.3/23.4	17.7/23.6	17.0/24.3	73.72	7670
3x2o	18.83–1.50	22.8/25.1	23.6/25.6	21.1/24.0	93.49	23109
3x2p	19.68–1.52	21.8/26.0	22.8/26.6	22.0/25.5	91.46	25101
4cvi	39.84–2.41	17.6/24.3	17.6/23.8	14.8/22.9	73.74	11426
4dvo	20.00–2.00	19.0/21.4	19.8/22.1	17.9/22.6	91.62	28663
4gpg	37.61–1.98	19.5/25.9	20.0/25.9	20.0/26.0	69.97	10446
4ny6	26.77–1.85	17.6/22.4	19.6/22.3	15.2/21.1	89.97	4654
4pdj	32.40–1.99	23.0/27.1	23.6/25.9	20.4/26.1	78.41	8315
4pvm	36.38–2.00	20.9/27.1	21.4/27.1	19.0/27.4	76.96	12062
4pvn	52.28–2.30	20.9/26.2	21.4/26.8	18.6/27.0	98.62	10831
4qcd	21.19–1.93	16.7/22.7	17.8/22.5	17.5/22.6	79.33	16540
4qdw	20.00–1.80	16.6/17.9	19.2/18.2	16.2/17.5	72.86	31459
4s2g	20.00–2.00	16.4/18.2	17.5/18.4	14.4/18.8	93.51	13125
4xpv	20.00–2.00	26.4/30.4	27.3/30.0	24.8/29.9	80.58	11251
5a93	15.03–2.20	21.7/23.6	23.0/22.8	15.9/23.7	71.94	11004
5cg5	53.57–2.40	18.6/22.9	19.3/22.9	17.5/22.8	98.45	17458
5cg6	22.12–2.40	26.0/28.7	26.0/25.3	24.2/27.0	98.25	17245
5jpr	36.71–2.20	23.6/31.0	23.6/31.0	20.2/31.2	68.13	8761
5mon	22.13–1.42	17.0/18.1	20.7/21.4	18.2/20.9	90.56	36196
5moo	22.09–1.43	17.0/18.5	19.9/20.9	18.0/20.3	93.83	36397
5mop	21.33–1.45	17.2/18.4	19.4/20.6	17.7/20.7	90.20	33722
5moq	25.43–1.50	15.0/16.7	17.1/18.5	15.3/18.5	89.39	30123
5mor	19.67–1.49	19.6/20.7	22.8/23.8	18.9/22.0	93.38	32293
5mos	22.15–1.50	16.6/18.0	18.7/19.5	16.9/19.8	88.77	30027
5xpe	17.02–2.09	22.5/27.8	24.2/28.2	21.0/28.7	78.13	9595
5zn0	33.76–1.90	18.8/24.7	19.6/24.9	17.4/25.6	97.05	22817
6bbr	15.00–2.30	22.1/24.6	25.8/25.1	21.4/26.1	90.97	7336
6bbz	15.43–2.20	19.8/22.0	24.2/24.0	19.7/24.6	92.18	8108
6bq8	40.00–2.20	23.2/28.8	22.8/28.4	20.5/30.6	72.77	5686
6exy	28.20–1.70	15.0/18.7	18.6/19.5	15.8/19.5	95.37	14401
6u0c	15.00–2.10	21.8/25.0	23.2/27.1	19.0/26.3	84.10	10060
6u0e	14.51–1.89	21.7/25.4	26.1/28.0	21.8/26.9	91.19	14921
6u0f	13.79–2.00	21.9/24.1	25.2/26.5	20.6/25.9	86.60	12087
7a0l	40.00–2.10	21.5/23.0	23.0/23.2	17.0/26.0	78.62	18131
7d6g	26.33–2.10	17.7/21.9	20.3/22.1	16.3/22.4	71.99	6598
7jun	14.96–2.50	20.1/25.3	20.3/25.2	17.9/26.5	83.14	7617
7tx3	14.12–1.89	22.6/27.5	23.0/27.9	21.8/28.5	93.70	22968
7tx4	12.75–2.35	17.7/25.9	17.9/25.9	16.4/26.1	85.83	5371
7tx5	32.87–2.30	18.7/25.9	19.4/26.5	18.1/27.1	75.39	5882

**Table 4:** Selected neutron models and corresponding X-ray reference models for re-refinement within *REFMAC*5 using external restraints. Comparison of published *R*-factor statistics and those obtained by re-refinement using *REFMAC*5.

PDB code		Published resolution range (low–high) (Å)		Published *R* values (work/free) (%)		*REFMAC*5 *R* values (work/free) (%)
Neutron	X-ray	Neutron	X-ray	Neutron	X-ray	Neutron refinement with external restraints
1c57	1dq6	15.79–2.40	8.00–1.90	27.0/30.1	18.6/NA	20.2/24.9
2efa	1b2a	80.00–2.50	55.00–1.70	21.6/29.1	18.8/23.0	21.9/25.3
2yz4	1dq6	33.64–2.20	8.00–1.90	27.9/31.2	18.6/NA	21.7/27.3
2zpp	1b2g	20.00–2.50	10.00–1.80	22.1/26.0	20.0/22.6	20.8/25.4
3r98	3r98	53.54–2.40	43.89–2.10	20.7/25.1	16.6/20.3	17.8/25.2
3r99	3r99	53.54–2.40	43.89–2.10	20.7/25.0	16.6/20.3	17.9/25.3
3vxf	3vxf	44.87–2.75	29.22–1.60	18.3/23.4	16.1/18.4	16.8/23.5
3x2o	3x2o	18.83–1.50	28.23–1.00	22.8/25.1	13.5/15.3	21.2/23.9
3x2p	3x2p	19.68–1.52	37.75–0.99	21.8/26.0	13.4/14.2	22.0/25.7
4cvi	4cvi	39.84–2.41	14.80–2.10	17.6/24.3	13.4/17.6	14.0/22.8
4dvo	4dvo	20.00–2.00	29.90–1.55	19.0/21.4	NA/NA	18.4/22.2
4gpg	4gpg	37.61–1.98	50.00–1.89	19.5/25.9	14.6/20.3	20.9/25.7
4pvn	4pvn	52.28–2.30	43.13–1.95	20.9/26.2	15.6/18.5	18.7/26.8
5cg6	5cg6	22.12–2.40	44.31–1.70	26.0/28.7	19.6/21.1	24.0/27.8
5xpe	5xpe	17.02–2.09	46.50–1.64	22.5/27.8	15.5/18.5	20.5/29.7
5zn0	5zn0	33.76–1.90	36.01–1.10	18.8/24.7	18.6/21.2	16.7/25.5
6bq8	6bq8	40.00–2.20	10.00–2.00	23.2/28.8	19.9/24.5	19.7/29.2
6exy	6exy	28.20–1.70	31.93–1.10	15.0/18.7	12.3/13.7	16.0/19.8
6u0e	6u0e	14.51–1.89	29.01–2.10	21.7/25.4	18.4/23.5	21.4/26.7
7d6g	7d6g	26.33–2.10	40.00–1.65	17.7/21.9	15.7/18.6	14.6/22.1
7tx4	7tx4	12.75–2.35	61.05–1.90	17.7/25.9	16.6/22.4	15.2/26.0
