# Supplementary material for: Neutron crystallographic refinement with REFMAC5 from the CCP4 suite
Source: Acta Crystallogr D Struct Biol. Author manuscript; Available in PMC 2024 Jan 17. (PMC7615533; doi:10.1107/S2059798323008793)
Supplement: Supplementary Tables [file EMS193351-supplement-Supplementary_Tables.pdf]

STRUCTURAL
BIOLOGY

Volume 79 (2023)

Supporting information for article:

Neutron crystallographic refinement with *REFMAC*5 from the *CCP*4 suite

Lucrezia Catapano, Fei Long, Keitaro Yamashita, Robert A. Nicholls, Roberto A. Steiner and Garib N. Murshudov

Table S1 Neutron data collection and re-refinement statistics for the UOX:8AZA complex

Data collection statistics	
Space group	*I*222
Temperature (°C)	20
Wavelength (Å)	3.12 – 4.20
Unit Cell (a, b, c; Å)	80.6, 96.1, 105.4
Resolution range (Å)[1]	40.00 – 2.10 (2.15 – 2.10)
CC ½ (%)[1]	98.7 (82.6)
< I/σ (I)[1]>	5.8 (2.9)
Completeness (%)[1]	78.62 (65.89)
Unique reflections[1]	18,131(1099)
Refinement statistics	
Resolution (Å)	40.00 – 2.10
R_{work}/R_{free} (%)	17.0 / 26.0
No. of reflections all/free	18,131/500
No. of	
Protein residues	295
Solvent molecules	350
Ligands	1
Ions	1
Average *B* factors (Å^2)	
Protein	45.18
Solvent	55.21
Ligand	36.23
Ion	44.00
r.m.s. deviations	
Bond lengths (Å)	0.006
Bond angles (°)	0.961

[1]High resolution statistics in parentheses.

Table S2 Neutron data collection and refinement statistics for the FutA structure

Data collection statistics	
Space group	$P2_1$
Temperature (°C)	21
Wavelength (Å)	3.10
Unit Cell (a, b, c; Å)	39.5, 78.3, 47.9
β angle (°)	97.4
Resolution range (Å)[1]	24.94 – 2.1 (2.18 – 2.1)
CC ½ (%)[1]	97.1 (68.1)
< I/σ (I)[1]>	4.8 (2.0)
Completeness (%)[1]	81.0 (52.6)
Multiplicity[1]	1.9 (1.1)
Unique reflections[1]	13,731 (890)
Refinement statistics	
PDB code	8OEN
Resolution (Å)	24.95 – 2.1
R_{work}/R_{free} (%)	18.2 / 25.0
No. of reflections all/free	13,731 / 698
No. of	
Protein residues	314
Solvent molecules	96
Ions	1
Average *B* factors (Å^2)	
Protein	22.31
Solvent	13.41
r.m.s. deviations	
Bond lengths (Å)	0.005
Bond angles (°)	1.142

[1]High resolution statistics in parentheses
